# Using network analysis to map the formal clinical reporting process in pediatric palliative care: a pilot study

**DOI:** 10.1186/1472-6963-11-343

**Published:** 2011-12-16

**Authors:** Harold Siden, Karen Urbanoski

**Affiliations:** 1 Department of Pediatrics, University of British Columbia, Child & Family Research Institute, 4480 Oak Street, Vancouver BC, V6H 3N1, Canada; 2 Centre for Addiction and Mental Health, Toronto ON, Canada

## Abstract

**Background:**

Continuity of care is a key component of care in complex and chronic conditions. Despite its importance, it is often absent in chronic-disease management. One challenge has been identifying tools to measure care continuity. In one context important to families, namely pediatric palliative care, we undertook a project to identify continuity and to pilot the use of network analysis as a tool.

**Methods:**

Network analysis studies patterns of relationships or interactions between members, providing qualitative and quantitative description of network structure.

**Results:**

In this report we applied network analysis to paper records of clinical consultations and reports for 6 patients with complex conditions. A high degree of discontinuity was identified, and care was fragmented amongst specialist and generalist providers. Information was shared selectively and often moved in only one direction.

**Conclusions:**

Families have anecdotally reported frustration with poor continuity of care. Network analysis can be a useful tool in describing the discontinuity of care experienced by families dealing with complex and chronic conditions. This tool could be expanded to other systems such as electronic health records and many other health care situations.

## Background

The term "continuity of care" in healthcare first appeared in the literature almost 80 years ago, and has been the subject of published research inquiry for at least 40 years [1,2]. Continuity of care can be thought of as the degree to which a series of discrete healthcare events are experienced as coherent and connected. As described in an important review by Haggerty, Reid, and colleagues, two core elements distinguish continuity of care from other attributes of care: a focus on experiences of the individual patient and consideration of the process of care over time [3]. Conceptually, continuity of care can be described and analyzed through three inter-related dimensions: informational, management, and relational. Each dimension is thought to contribute to the overall sense that a patient experiences consistency and coordination of their care, when it is provided by multiple health care practitioners or even multiple organizations.

Continuity of care is valued by patients and, by extension, parents caring for children with chronic health conditions [4,5]. Continuity of providers is a major element of patient and parent satisfaction with care [6] and has been linked to improved care processes [7]. There is, however, a dearth of research developing and testing approaches to support continuity of care for children with chronic diseases. To this aim, studies are needed to enrich our understanding of continuity in pediatric care from multiple perspectives (e.g., parents, other formal and informal caregivers, health professionals) and across the multiple dimensions named above.

From a methodological standpoint, continuity of care is a complex construct that can be evaluated in a number of different ways. In a recent study, Miller, Condin and colleagues successfully used qualitative methodology to obtain rich and powerful narratives of parents' perspectives of continuity of care for their children with chronic health conditions [8]. Parents' experiences coalesced with Haggerty and Reid's dimensional framework of continuity of care. The narratives also identified points of fragmentation and compartmentalization of services, and described the frustration of (some) parents at needing to step in to coordinate their child's care.

These findings also agree with the clinical experience of one of the present authors (HS), which more generally provided the impetus for the present work. Specifically, the families of children diagnosed with life-limiting conditions often voice frustration at not having a "map" to guide them as they navigate the array of required services. The clinical context for care of children with severe chronic illnesses typically involves a wide variety of providers and agencies from an array of disciplines and sectors. As such, the salience of inter-provider and inter-disciplinary communication and coordination may be especially heightened. To the aim of furthering our understanding of continuity of care in this setting, we conducted a pilot study to evaluate the utility of a specific methodology, namely network analysis, in mapping out the process of information sharing/continuity. We applied the network analysis method to study how clinical reports are shared as one aspect of informational continuity.

Network analysis involves the study of patterns of relationships or interactions between members, providing both qualitative and quantitative description of network structure and supporting further study of network functioning [9]. Analysis of social networks has a strong tradition, and focuses on the ties between nodes (e.g., individuals), how those connections occur and their strength; the analysis is now aided by new theories and techniques. Recent examples of its application in health care include studies of interactions and the flow of information between providers in primary care settings [10], mental health service agencies [11], and organizations at broader system-levels [12]. Network data, that is, the ties between member individuals or agencies, can be derived in a number of ways (e.g., from specially designed surveys, structured interviews, field notes) [10]. Secondary to the objective of determining the general utility of this methodological approach, this pilot study addressed the utility of using chart review to gather network data on the formal, written communication process.

## Methods

### Data collection

The research protocol was approved by the institutional review boards at the University of British Columbia, Children's and Women's Health Centre of British Columbia (BC), and Canuck Place Children's Hospice, all in Vancouver, Canada. Data were collected through retrospective chart review of paediatric patients who had been diagnosed with life-limiting conditions (n = 18). The subjects, chosen at random from a larger study sample, were referred for or receiving care through BC Children's Hospital and Canuck Place Children's Hospice between January and March of 2008. Each child's hospice and hospital chart was reviewed for the 3-month period following their most recent hospice visit.

Clinicians and patients were assigned unique identifying codes to preserve anonymity, and de-identified data were obtained via chart review. Relevant data collected from patient files included basic demographic information, diagnostics, lab/test history, and involvement with health care professionals. Data were entered into an electronic spreadsheet to facilitate analysis.

### Data analysis

This pilot phase focused specifically on the written communications that occurred between care providers during the 3-month study period. The patient chart serves as a record of this communication and can be used to determine the presence and frequency of interaction between the providers and agencies that comprise a patient's care network. The care network was first defined for each patient, including the individual clinicians, clinical teams, and agencies who sent, received, or were copied on letters or reports found in the patient chart, or who were identified as being involved in the patient's care during the hospice intake process. Each of the network members was assumed to have the potential of receiving, recording, and relaying information; however, it was also expected that one or more family physicians or pediatricians would play a central role in network communications. Residents, medical students, and auxiliary staff were not included in the analysis due to a high rotation rate and the expectation that a senior physician was managing the care centrally. Of the 18 original subjects, 6 had a sufficient number of reports or letters recorded in their charts over the 3 months to be suitable for analysis.

Matrices were constructed to denote the flow of written information (e.g., letters, reports) between network members. A tie between network members represents the presence of a written communication documented in the patient chart, indicating that the provider sent, received, or was copied on a letter or report from another network member. The majority of reports or letters were actually sent to the patients' charts (i.e., they were not explicitly addressed to any specific provider); however, they were typically copied to one or more other providers. The analysis preserved both the direction and frequency of communications. The relationships were treated as asymmetrical (i.e., reciprocity of communication was not assumed unless specifically indicated).

The matrices were diagrammed to provide a visual representation of communications between network members. These diagrams can be visually examined to describe the structure of the network, including the density of relationships between network members and the presence of holes or disconnects, which indicate potential breaks in the flow of information. The direction of communication is indicated by arrowheads, while the frequency of communication is denoted by line thickness (i.e., thicker lines indicate a greater number of communications between a given pair of providers). Each network member was also characterized in terms of its centrality within each patient's care network. Degree centrality quantitatively describes each network member's position or importance within the network, by summarizing the number of ties sent out and received from other members of the network. The analysis was conducted with specialized network software, UCINET v.6 [13] and NetDraw v.2 [14].

## Results

**Figure 1:**
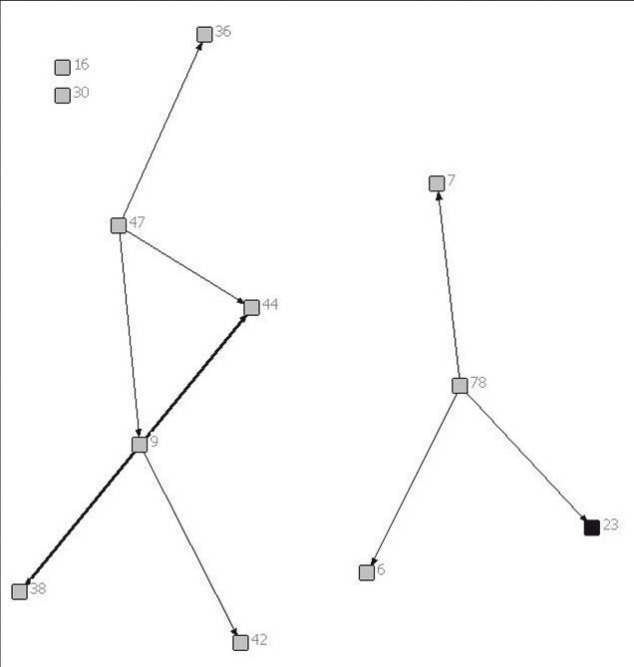
**Network diagram for patient 1**.

**Figure 2:**
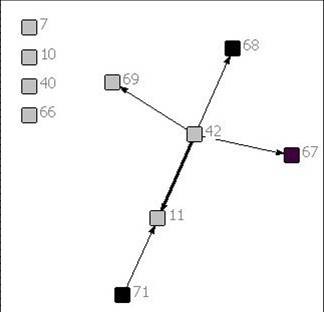
**Network diagram for patient 2**.

**Figure 3:**
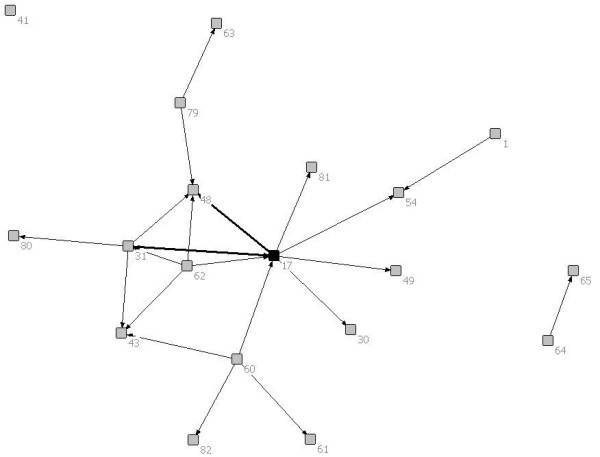
**Network diagram for patient 3**.

**Figure 4:**
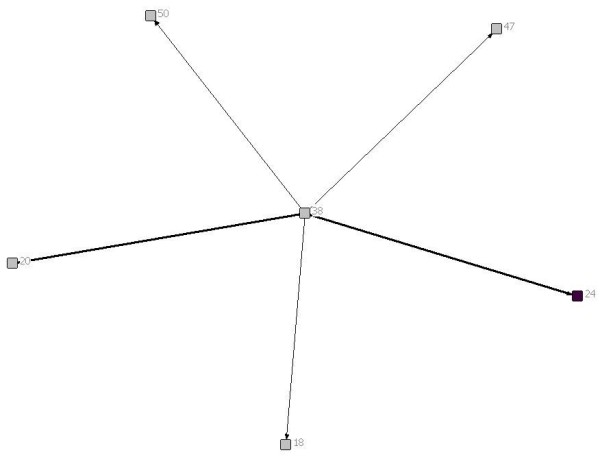
**Network diagram for patient 4**.

**Figure 5:**
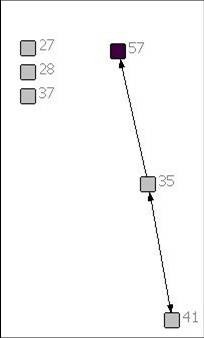
**Network diagram for patient 5**.

**Figure 6:**
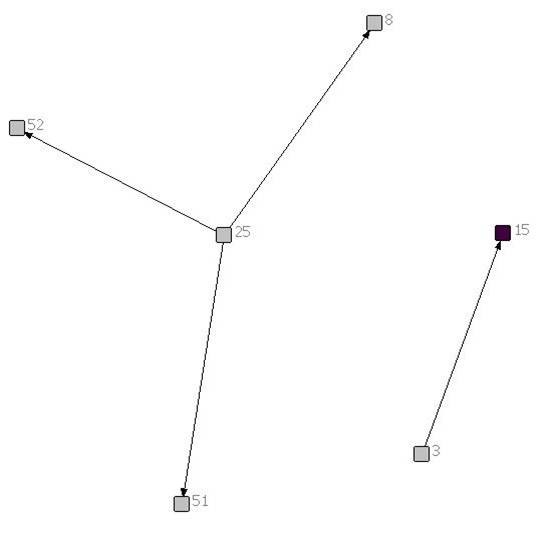
**Network diagram for patient 6**.

Of the 6 patients included in this report, 2 had brain tumours, 1 had a solid tumour, 1 had a neuromuscular disease, 1 had a congenital heart defect and 1 had a neuro-metabolic condition. Figures 1, 2, 3, 4, 5 and 6 depict the care networks constructed for each patient. These are accompanied by Tables 1, 2, 3, 4, 5 and 6, which summarize degree centrality (i.e., communications sent out and received) for each member of the 6 networks. The tables also include information on provider type and the mean number of ties per network member. Family physicians and pediatricians are highlighted in the diagrams and tables because of their hypothesized central role in the networks.

**Table 1:** Degree centrality for patient 1

Health professional/service		Communications sent out	Communications received
6	Oncologist	0	1
7	Psychologist	0	1
9	Neurosurgeon	5	1
16	Primary nurse, Oncology	0	0
**23**	**Family Physician**	**0**	**1**
30	Pediatrician/Palliative Care	0	0
36	Oncologist	0	1
38	Oncologist	0	2
42	Pediatrician/Palliative Care	0	1
44	Neurosurgeon	0	3
47	Neurosurgeon	3	0
78	Canuck Place	3	0
	*Mean*	*0.92*	*0.92*

**Table 2:** Degree centrality for patient 2

Health professional/service		Communications sent out	Communications received
7	Psychologist	0	0
10	Primary Nurse	0	0
11	Oncologist	0	3
**40**	**Family Physician**	**0**	**0**
42	Pediatrician/Palliative Care	5	0
66	Hematologist/Oncologist	0	0
**67**	**Family Physician**	**0**	**1**
**68**	**Family Physician**	**0**	**1**
69	Ministry of Children and Family Development	0	1
**71**	**Family Physician**	**1**	**0**
	*Mean*	*0.60*	*0.60*

**Table 3:** Degree centrality for patient 3

Health professional/service		Communications sent out	Communications received
1	Surgical Nurse	1	0
**17**	**Pediatrician**	**8**	**3**
30	Pediatrician/Palliative Care	0	1
31	Otolaryngologist	4	3
41	Pediatric Neurologist	0	0
43	Family Physician	0	3
48	Pediatrician/Biochemical Diseases	0	5
49	Social Worker	0	1
54	General Surgeon	0	2
60	Physiotherapist/Internal Case Coordinator	4	0
61	External Case Coordinator	0	1
62	Audiologist	4	0
63	Pediatrician	0	1
64	Pediatric Consultant/Visual Impairment	1	0
65	Ophthalmologist	0	1
79	Biochemical Lab	2	0
80	Audiology Lab	0	1
81	At-home program/Occupational Therapist	0	1
82	Parents	0	1
	*Mean*	*1.26*	*1.26*

**Table 4:** Degree centrality for patient 4

Health professional/service		Communications sent out	Communications received
18	Radiation Oncologist	0	1
20	Pediatric Rheumatologist	0	2
**24**	**Family Physician**	**0**	**2**
38	Oncologist	7	0
47	Pediatric Neurosurgeon	0	1
50	Nurse, Oncology	0	1
	*Mean*	*1.17*	*1.17*

**Table 5:** Degree centrality for patient 5

Health professional/service		Communications sent out	Communications received
27	Pediatric Dentist	0	0
28	ER Physician	0	0
35	Pediatric Dermatologist	2	1
37	Orthopaedic Surgeon	0	0
41	Pediatric Neurologist	1	1
**57**	**Family Physician**	**0**	**1**
	*Mean*	*0.50*	*0.50*

**Table 6:** Degree centrality for patient 6

Health professional/service		Communications sent out	Communications received
3	Medical Geneticist	1	0
8	Pediatrician/Palliative Care	0	1
**15**	**Family Physician**	**0**	**1**
25	Cardiologist	3	0
51	Pediatrician	0	1
52	CHN	0	1
	*Mean*	*0.67*	*0.67*

The care networks ranged in size from 6 members for patients #4, #5 and #6, up to 19 members for patient #3. All were multidisciplinary in nature and all contained either a family physician or primary pediatrician. However, great variation in network composition was evident. For instance, while the network for patient #1 included 3 oncologists and an oncology nurse, 3 neurosurgeons, 2 palliative care pediatricians, a psychologist, the Canuck Place hospice team, and 1 family physician (Table 1), the network for patient #2 included 4 family physicians, 2 oncologists, a psychologist, a primary nurse, and the Ministry of Children and Family Development (Table 2). The care network for patient #3 was the most extensive and included the broadest array of services and supports (Table 3). It was also the sole care network in the study to explicitly include the parents in the formal clinical reporting process.

The number of reports sent or received in the 3 months ranged from 2 for each of patients #4, #5 and #6, up to 11 reports for patient #3. Across the six patients, a total of 25 reports were located. Of these, 18 were sent to the patient's chart, typically copying one or more specific care provider (range 1-5). However, 2 reports (for patients #2 and #3) were simply placed in the patient's chart without being addressed to any other providers. Without their being explicitly copied, it is not possible to confirm whether the other providers involved in the patient's care would have had reliable access to the report and would have been aware of its findings.

Overall, the number of ties between network members was quite low, in most cases averaging less than 1 per provider. Exceptions were the care networks for patients #3 and #4, where the number of ties averaged 1.26 and 1.17 per provider (Tables 3 and 4). Particularly for patient #3, whose network contained 19 members, this average is still low. With the exception of the network for patient #4 (Figure 4), all of the networks contained providers who were identified as members of the care team, but who were not included or copied on any of the reports or letters passing between the other members during the study period. The networks for patients #1 and #6 were split into two separate parts that did not explicitly communicate with each other during this time. This can be contrasted with the network for patient #4, in which all communications passed through one individual, an oncologist (38; Figure 4). No communication was documented between the other members in the network.

In no care network was a family physician the most active in terms of sending or receiving information (i.e., as measured by degree centrality). Across networks, the most active and central members were specialists in neurosurgery (patient #1), pediatrics (patients #2 and #3), oncology (patients #2 and #4), dermatology (patient #5), or cardiology (patient #6). Of particular note, in the care network for patient #3, one pediatrician (17; Figure 3) stood out as a central member, both initiating and receiving a fair number of written communications. This provider was involved in 7 of the 11 reports that were circulated during the study period (i.e., sending out 4 reports, each of which was copied to 1 to 3 other providers, and being copied on 3 additional reports). Through written documentation, therefore, this provider was aware of much of the care-related activity concerning this patient. However, there was also evidence of active communication between other members of the network. For instance, a second pediatrician (48) was also kept apprised of much of the care-related activity for this patient, being copied on 5 of the 11 reports. In addition, the otolaryngologist (31) was active in both sending and receiving information, sending reports out to 4 other providers and receiving 3 him- or herself. Finally, the physiotherapist (60) and audiologist (62) each sent out reports out to 4 other network members (Table 3).

Aside from this example, however, reciprocity in formal communications was typically low across these networks, meaning that those who sent out or copied others on their reports were often not the same network members who received information back in turn. As an example, in the network for patient #2, the pediatrician (42; Figure 2) who was most active in sending out ties was not copied in turn on the remaining communication that occurred during the study period.

## Discussion

In this pilot project, we sought to determine the general potential of network analysis for evaluating patterns of communication between health care providers - an important aspect of continuity of care - and, more specifically, the feasibility of mapping out the formal clinical reporting process using data obtained from patient records. Overall, these findings suggest that network methods show promise as a tool for evaluators concerned with questions of care continuity. The relational perspective brought by network analysis has value for health services researchers and planners in the evaluation of different patterns and modalities of communication and other program interventions. Specifically, it provides a straight-forward way of both describing and testing hypotheses about patterns of communication by patient characteristics (e.g., diagnosis, socio-economic status, outcomes) and provider characteristics (i.e., care setting, specialty, years in practice). Findings may support efforts to develop and test models of optimal communication patterns, which can in turn inform ongoing work on continuity of care and the link to patient outcomes.

At this preliminary stage, the substantive conclusions emanating from this research are limited. From the perspective of formal clinical reporting, these care networks were typically fragmented and characterized by a low degree of document-sharing and reciprocity. In addition, there was also variability evident in the density and reciprocity of the formal clinical reporting process across the care networks for individual patients. These findings await replication in larger samples, but provide initial support for the use of network analysis to explore patterns of communication and link them to patient outcomes. Notably, this work complements that by Miller *et al *qualitatively describing parents' experience of continuity of care (or lack thereof) [8]. Together, our study and that by Miller *et al *explore a very broad range of serious, chronic, childhood conditions, ranging from mental health conditions (Attention Deficit Hyperactivity Disorder), to diseases with long-term health needs (spina bifida), and then to conditions ending in early death (neurodegenerative diseases). It is across this broad spectrum that continuity of care needs to be improved. Whether there are differences in the experience of continuity of care across broad diagnostic categories with varying co-occurring conditions is an interesting avenue for future study.

In this pilot study, we examined the capabilities of network analysis with respect to a single aspect of informational continuity: written communications. The importance of written documentation as a necessary component of informational continuity has been noted, particularly in clinical environments characterized by low relational continuity (i.e., when there is variability and change in the involvement of particular providers over time) [8]. Written documents provide evidence of communication and support long-term information retention. As such, it constituted an appropriate starting point for evaluation. However, written communications provide only one (formal and conservative) channel for the flow of information through a network, and are not sufficient to alone ensure informational continuity. At the sites used in this study, an electronic health record is not implemented, nor is there a secure email system outside the hospital which would extend to community providers. Therefore, while this analysis cannot claim to have identified all of the communication activity that took place during the study period, it provided an initial indication of likely places that communication falls apart over time. For instance, the general lack of involvement of family physicians and pediatricians in the formal clinical reporting process across the care networks presented here is a potential point of concern.

Over and above the written documentation that ends up in patient charts, broader assessment of informational continuity through primary collection of network data (e.g., via surveys or structured interviews with network members) would be highly valuable. Use of chart review precluded consideration information flow via email, telephone, and in-person conversations. The extent to which these forms of communication play a role in the care of chronically-ill children, and their relative effectiveness in relaying important information, is unknown. The degree to which providers mix and match communication modalities and technologies is a worthwhile inquiry for future investigations.

This pilot study shows the potential of using the framework of a network to better understand health care provision. The next step will be to expand primary data collection in order to undertake an evaluation of a broader set of indicators of network structure, such as provider perceptions of the helpfulness or value of collaboration with other network members, or provider awareness of the service mandates and details of care provided by other network members. This would provide valuable contextual information for mapping the formal clinical reporting process, and interpreting network diagrams and meaning of centrality indicators. Classes of providers grouped according to their centrality across a number of measures of network structure can be generated statistically (i.e., using cluster analysis), and linked with traditional attributional data (e.g., patient outcomes, satisfaction with care, provider characteristics) to examine questions of network functioning. In addition, the qualitative and quantitative indicators of network structure could be used to inform and supplement additional qualitative investigation of parent or other caregiver perceptions of continuity; for instance, by asking parents to comment on the meaning of identified patterns of communication between providers and interpreting the placement of different providers within the care network. They could equally be used in longitudinal evaluations to determine change in structure following the introduction of specific interventions designed to improve continuity of care.

The network approach can successfully capture elements of direction, frequency and reciprocity in the flow of information. It lacks, however, the capability to address the time-sequence inherent in provider interactions. It could, therefore, be helpfully supplemented by methods such as process mapping, which are able to capture the sequence and time-dependency of interactions in documenting continuity [15,16]. A team of Canadian researchers has successfully applied process mapping techniques to describe the journey of patients through the health care system [17-19].

## Conclusions

In summary, we believe our findings in this pilot study highlight the promise of network approaches to the study of continuity of care among chronically-ill children, and likely to chronic disease management in general. As part of a mixed-method approach to the study of continuity of care, and supplemented by attributional data and indicators of time-sequence, the analysis of relational data with network methods offers a valuable depiction of interactions and ties in complex clinical contexts. It provides a straight-forward way of identifying the occurrence and placement of gaps in informational continuity, and to identify particular providers who occupy central and influential roles within specific care networks. The mix of quantitative and qualitative information on network structure can be easily integrated with additional contextual data, as described above.

We hope that this pilot study will be the starting point for a fuller, richer analysis with mixed methods design to describe the nature of care as experienced by children with chronic disease. Ultimately, a comprehensive understanding of this care process may provide the basis for new program initiatives to make care more efficient, effective, safe and satisfying overall.

## Competing interests

The authors declare that they have no competing interests.

## Authors' contributions

The authors contributed equally to this work. HS developed the research question and gathered the case examples. KU undertook the network analysis. Both authors contributed equally to writing the manuscript. Both authors read and approved the final manuscript.

## Pre-publication history

The pre-publication history for this paper can be accessed here:

http://www.biomedcentral.com/1472-6963/11/343/prepub
